# Comparison of outcomes of KPC-producing Enterobacterales bloodstream infections treated with ceftazidime-avibactam and other conventional antibiotics: retrospective single-center study

**DOI:** 10.1128/spectrum.02819-25

**Published:** 2026-03-12

**Authors:** Kyungsup Kwon, So Yun Lim, Euijin Chang, Seongman Bae, Min Jae Kim, Yong Pil Chong, Sung-Han Kim, Sang-Ho Choi, Sang-Oh Lee, Yang Soo Kim, Jiwon Jung

**Affiliations:** 1 Department of Infectious Diseases, Asan Medical Center, University of Ulsan College of Medicine 37994, Seoul, Republic of Korea; Emory University School of Medicine, Atlanta, Georgia, USA

**Keywords:** carbapenemase-producing Enterobacterales, *Klebsiella pneumoniae *carbapenemase, ceftazidime-avibactam

## Abstract

Ceftazidime–avibactam (CZA) was introduced in South Korea in July 2023, but regional data on its clinical efficacy, treatment outcomes, and microbiological characteristics of bloodstream infections (BSIs) caused by *Klebsiella pneumoniae* carbapenemase (KPC)-producing Enterobacterales remain limited. This study evaluated the effectiveness of CZA compared with conventional therapies targeting KPC-producing Enterobacterales BSIs. A retrospective observational study of adult patients with KPC-producing Enterobacterales BSIs was conducted at a tertiary care center in Seoul, Republic of Korea. Patients treated with CZA between July 2023 and November 2024 were compared with a historical cohort treated with best available agents (conventional therapy) from December 2021 to June 2023. The primary endpoint was 30-day all-cause mortality. Secondary endpoints included BSI persistence, BSI recurrence, and emergence of resistance. A total of 106 patients received CZA and 156 received best available agents. The 30-day mortality rate was significantly lower in the CZA group compared with the comparator group (15.1% vs 28.2%, *P* = 0.02). Persistent BSI was also significantly reduced in the CZA group (5.0% vs 34.2%, *P* < 0.001). In multivariate analysis, CZA therapy was independently associated with reduced 30-day mortality (adjusted OR, 0.46; 95% CI, 0.22–0.92). Kaplan–Meier analysis demonstrated improved survival with CZA (*P* = 0.014), and CZA resistance emerged in three patients (2.8%, 3/106). In this Korean cohort, CZA therapy was associated with significantly lower 30-day mortality and persistent BSI rates in patients with KPC-producing Enterobacterales BSIs, compared with best available agents.

**IMPORTANCE:**

Bloodstream infections caused by *Klebsiella pneumoniae* carbapenemase (KPC)-producing Enterobacterales carry high mortality rates and offer few treatment options, representing an urgent public health threat. Ceftazidime–avibactam (CZA) has emerged as a key therapy for these infections globally, yet real-world clinical data from South Korea have been absent since its recent introduction. This study presents the first comprehensive evaluation of CZA for treating KPC-producing Enterobacterales bloodstream infections in a Korean tertiary-care hospital. Through direct comparison with conventional combination regimens, we show that CZA substantially reduces 30-day mortality and persistent bacteremia. These findings provide important evidence for CZA as an effective first-line treatment, especially in settings where KPC-producing organisms are highly prevalent. Our results emphasize the value of timely access to novel β-lactam/β-lactamase inhibitors and point to the ongoing need for resistance surveillance.

## INTRODUCTION

Multidrug-resistant organisms have emerged as a major global health threat, contributing to an estimated 5 million deaths annually worldwide (1). Among them, carbapenem-resistant Enterobacterales (CRE) have been designated critical priority pathogens by the World Health Organization (WHO) (2). Of particular concern are carbapenemase-producing Enterobacterales (CPE), as their enzyme-mediated resistance facilitates transmission, limits treatment options, and increases healthcare burden (2, 3). In a nationwide study from South Korea, CRE bacteremia was associated with a 90-day mortality rate of 55.0% and an estimated annual socioeconomic burden of approximately 45.9 million USD (4). Although carbapenems were previously the mainstay of treatment for multidrug-resistant Gram-negative infections, the rise of CPE has severely restricted effective therapeutic options. Ceftazidime–avibactam (CZA), a novel β-lactam/β-lactamase inhibitor combination, demonstrates potent activity against *Klebsiella pneumoniae* carbapenemase- (KPC)- and OXA-48-producing organisms and is regarded as a key therapeutic agent in treating such infections (5).

Since the establishment of a national surveillance system for CRE in 2017, South Korea has seen an annual 20%–30% increase in CRE infections, with KPC producers comprising 77.4% of CPE isolates (6). Although CZA was approved by the U.S. Food and Drug Administration (FDA) in 2015, it was not introduced in South Korea until July 2023 (7). *In vitro* studies conducted locally have reported CZA susceptibility rates exceeding 90%; however, clinical data on real-world outcomes remain limited (8, 9). This study, therefore, aimed to evaluate the clinical effectiveness of CZA compared with conventional antibiotic regimens in patients with bloodstream infections (BSIs) caused by KPC-producing Enterobacterales at a tertiary care center in South Korea.

## MATERIALS AND METHODS

### Study design and setting

A retrospective observational cohort study was conducted at a single tertiary care hospital in Seoul, South Korea. As CZA became available in July 2023, patients with BSIs caused by KPC-producing Enterobacterales were routinely treated with CZA. The CZA group comprised patients treated with CZA between July 2023 and November 2024. The comparator group included patients treated with best available agents (conventional CPE-targeted therapy) between December 2021 and June 2023. For patients with multiple episodes of BSI during the study period, only the first (index) culture was included. The inclusion criteria were as follows: (i) individuals aged ≥ 18 years; (ii) at least one positive blood culture yielding KPC-producing Enterobacterales; (iii) receipt of the relevant antibiotic therapy for ≥48 h, defined as either best available agents or CZA as described below.

The primary outcome was 30-day all-cause mortality, defined as death from any cause within 30 days of the index-positive blood culture. Secondary outcomes included: persistent BSI, defined as failure to clear the BSI (i.e., persistence of KPC-producing Enterobacterales in follow-up blood cultures despite ≥48 h of appropriate therapy); recurrent BSI, defined as a new episode of BSI caused by KPC-producing Enterobacterales within 100 days after completion of the index treatment; and emergence of resistance to CZA, defined as isolation of a KPC-producing organism with a newly elevated minimum inhibitory concentration (MIC) for CZA above the susceptible breakpoint, either during therapy (breakthrough infection) or upon recurrence (acquired resistance). 90-day all-cause mortality was also assessed as a longer-term outcome.

### Definitions

Best available agents were defined as any regimen containing tigecycline, aminoglycosides, colistin, or carbapenems administered as part of combination therapy. The primary source of infection was classified according to the Centers for Disease Control and Prevention (CDC) criteria, described by Horan et al. (10). Bloodstream infections were categorized as follows: community-acquired, defined as a positive blood culture obtained ≤48 h after hospital admission in patients without prior healthcare exposure; hospital-acquired, defined as an infection developing ≥48 h after hospital admission; and healthcare-associated community-onset, defined as a positive blood culture obtained within 48 hours of admission with healthcare-associated risk factors, as described by Friedman et al. (11). Per institutional protocol, follow-up blood cultures were obtained every 48 hours in patients with persistent fever or positive cultures. Duration of bacteremia was calculated from the index positive culture to the first negative culture drawn ≥48 h after the last positive culture.

### Microbiological data

Blood culture isolates were identified to the species level, and antimicrobial susceptibility testing was performed using the MicroScan WalkAway 96 Plus system with the Neg Combo Panel Type 72 (Beckman Coulter, Brea, CA, USA). Interpretations followed the latest Clinical and Laboratory Standards Institute (CLSI) guidelines. For colistin, MICs were determined using the BD Phoenix M50 ID/AST system after internal verification (data not shown) and interpreted according to CLSI M100, 34th ed. (Intermediate ≤2 mg/L; Resistant ≥4 mg/L; no susceptible category). Reference broth microdilution testing was not routinely performed. All CRE isolates were screened for carbapenemase production using phenotypic methods, including (i) the modified Hodge test and (ii) a carbapenemase inhibition assay using phenylboronic acid and ethylenediaminetetraacetic acid (EDTA) (12). Genotypic confirmation was performed using a multiplex real-time PCR assay (Xpert Carba-R) with gene-specific primers. Susceptibility testing and breakpoint interpretations for CZA followed CLSI performance standards (13).

### Statistical analysis

Baseline characteristics and clinical outcomes were compared between the CZA and comparator groups. Categorical variables were analyzed using the Chi-square or Fisher’s exact test, and continuous variables using the Mann–Whitney *U* test for non-parametric data. Kaplan–Meier survival curves were constructed for 30-day survival, with comparisons made using the log-rank test. Univariate logistic regression was performed to identify risk factors for 30-day mortality. Variables with *P* < 0.05 in univariate analysis were included in the multivariable logistic regression model. Adjusted odds ratios (aOR) and 95% confidence intervals (*CIs*) were reported from the multivariable model. Propensity score matching was conducted as a sensitivity analysis to reduce residual confounding between groups. We performed 1:1 nearest-neighbor matching using a caliper of 0.2, targeting standardized mean differences (SMDs) < 0.1 after matching. In the matched cohort, we estimated the association between treatment group and 30-day all-cause mortality using conditional logistic regression and reported odds ratios (ORs) with 95% *CIs*. All tests were two-tailed, with a *P*-value of < 0.05 considered to indicate statistical significance. Analyses were conducted using R software (version 4.4.1).

## RESULTS

### Clinical characteristics of cohort patients

**Fig 1:**
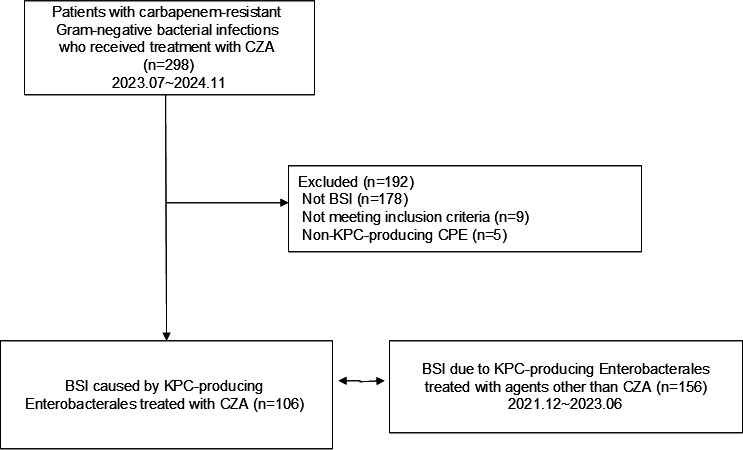
Study cohort selection for KPC-producing Enterobacterales bloodstream infections treated with or without CZA. BSI, bloodstream infection; CZA, ceftazidime-avibactam.

A total of 262 patients with KPC-producing Enterobacterales BSI were included: 106 (40%) in the CZA group and 156 (60%) in the comparator group (Fig. 1). The demographic and clinical characteristics of the two groups are summarized in Table 1. No significant differences were observed in baseline characteristics, including age, ward of admission, infection acquisition category, Pitt bacteremia score, and Charlson Comorbidity Index. However, the comparator group included a higher proportion of male patients (56.4% vs 46.2%, *P* = 0.003). Conversely, hematologic malignancy (39.6% vs 25.6%, *P* = 0.02), documented CPE colonization at the time of the index culture (84.0% vs 63.5%, *P* = 0.001), and catheter-related BSI as the primary source (15.1% vs 7.1%, *P* = 0.04) were more common in the CZA group. Prior exposure (within 3 months) to aminoglycosides, tigecycline, and carbapenems was significantly more frequent in the CZA group, whereas third- or fourth-generation cephalosporins were more often used in the comparator group (all *P* ≤ 0.02). Solid tumors (42.3% vs 28.3%, *P* = 0.03) and chronic heart failure (11.5% vs 1.9%, *P* = 0.01) were more prevalent in the comparator group. No other statistically significant differences were observed.

**TABLE 1:** Demographic and clinical characteristics of patients treated with CZA versus best available agents^*a*^

Characteristics	CZA treatment (*n* = 106)	Best available agents, comparator (*n* = 156)	*P*-value
Age (years), median (IQR)	63.0 (56.0–70.0)	64.0 (56.0–70.0)	0.85
Male sex	49 (46.2)	102 (65.4)	0.003
Ward at index culture			
Medical (all)	56 (52.8)	89 (57.1)	0.50
Hematology	32 (30.2)	34 (21.8)	0.12
Surgical (all)	27 (25.5)	39 (25.0)	0.93
Transplant	26 (24.5)	27 (17.3)	0.15
ICU	23 (21.7)	28 (17.9)	0.45
Acquisition site			0.56
Community	1 (0.9)	1 (0.6)	
Healthcare-associated community	18 (17.0)	20 (12.8)	
Healthcare	87 (82.1)	135 (86.5)	
Previous CPE bacteremia	12 (11.3)	8 (5.1)	0.11
Organism			
*Klebsiella pneumoniae*	105 (99.1)	148 (94.9)	0.09
*Escherichia coli*	1 (0.9)	8 (5.1)	0.09
*Klebsiella oxytoca*	0 (0.0)	1 (0.6)	>0.999
CPE colonization at index culture	89 (84.0)	99 (63.5)	0.001
Site of infection			
Intra-abdominal	40 (37.7)	56 (35.9)	0.79
Biliary tract	37 (34.9)	62 (39.7)	0.44
Catheter-related	16 (15.1)	11 (7.1)	0.04
Pneumonia	7 (6.6)	13 (8.3)	0.64
Skin and soft tissue	1 (0.9)	6 (3.8)	0.25
Urinary tract	4 (3.8)	7 (4.5)	>0.999
Primary bacteremia	1 (0.9)	1 (0.6)	>0.999
Pitt bacteremia score, median (IQR)	1.0 (0.0–2.0)	1.0 (0.0–3.0)	0.79
Charlson comorbidity index, median (IQR)	5.0 (4.0–7.0)	6.0 (4.0–8.0)	0.97
Time to appropriate treatment, median (IQR)	2.0 (0.0–3.0)	2.0 (1.0–4.0)	<0.001
Comorbidities			
Chronic liver disease	43 (40.6)	51 (32.7)	0.24
Hematological malignancy	42 (39.6)	40 (25.6)	0.02
Neutropenia	36 (34.0)	39 (25.0)	0.15
Diabetes mellitus	35 (33.0)	43 (27.6)	0.42
Solid organ transplant	32 (30.2)	40 (25.6)	0.50
Solid cancer	30 (28.3)	66 (42.3)	0.03
Chronic kidney disease	15 (14.2)	24 (15.4)	0.92
Cerebrovascular accident	7 (6.6)	7 (4.5)	0.64
Hematologic stem cell transplant	6 (5.7)	14 (9.0)	0.45
Chronic pulmonary disease	2 (1.9)	11 (7.1)	0.11
Chronic heart failure	2 (1.9)	18 (11.5)	0.01
Mechanical ventilation at index culture	24 (22.6)	31 (19.9)	0.70
CRRT at index culture	13 (12.3)	10 (6.4)	0.16
ECMO at index culture	1 (0.9)	3 (1.9)	0.65
Antibiotic use within 3 months			
β-Lactam with BLI	63 (59.4)	92 (59.0)	>0.999
First- and second-generation cephalosporin	12 (11.3)	26 (16.7)	0.30
Third- and fourth-generation cephalosporin	49 (46.2)	97 (62.2)	0.02
Aminoglycoside	62 (58.5)	12 (7.7)	<0.001
Quinolone	49 (46.2)	58 (37.2)	0.18
Tigecycline	34 (32.1)	14 (9.0)	<0.001
Carbapenem	82 (77.4)	99 (63.5)	0.02
Glycopeptide	51 (48.1)	86 (55.1)	0.32
Colistin	2 (1.9)	8 (5.1)	0.21
Procedure before index culture			
Surgery within 1 month	13 (12.3)	23 (14.7)	0.70
Dialysis within 1 month	7 (6.6)	17 (10.9)	0.34
Endoscopy within 1 month	12 (11.3)	24 (15.4)	0.45
Indwelling catheter	84 (79.2)	146 (92.9)	0.002
Outcome			
30-day mortality	16 (15.1)	44 (28.2)	0.02
90-day mortality	28 (26.4)	67 (42.9)	0.01
Persistent BSI	5 (5.0)^*b*^	51 (34.2)^*c*^	<0.001
Duration of bacteremia, median (IQR)	3.0 (2.0–5.0)^*d*^	5.0 (3.0–10.0)^*c*^	<0.001
Recurrent BSI within 100 days	14 (13.2)	19 (12.2)	0.96
Breakthrough infection	1 (0.9)	–^*e*^	
Recurrent infection with acquired resistance	2 (1.9)	–	

^*a*^ Data are presented as *n* (%) unless otherwise indicated. BLI, β-lactamase inhibitor; BSI, bloodstream infection; CPE, carbapenemase-producing Enterobacterales; CRRT, continuous renal replacement therapy; ECMO, extracorporeal membrane oxygenation; ICU, intensive care unit; IQR, interquartile range.

^*b*^ Data were missing for four patients.

^*c*^ Data were missing for seven patients.

^*d*^ Data were missing for six patients.

^*e*^ –, not available.

A detailed comparison of baseline clinical characteristics between solid organ transplantation (SOT) and non-SOT patients is presented in Table S1. SOT recipients were significantly younger than non-SOT patients (median age 61.0 [IQR, 55.0–64.5] vs 66.0 [IQR, 57.0–71.0] years, *P* < 0.001). Notably, SOT patients had significantly higher rates of ICU admission (31.9% vs 14.7%, *P* = 0.003), hemodialysis (22.2% vs 8.4%, *P* = 0.005), and continuous renal replacement therapy (CRRT) (15.3% vs 6.3%, *P* = 0.04). A trend toward earlier initiation of appropriate antibiotics within 24 h was observed in SOT patients (48.6% vs 35.8%, *P* = 0.058). Other characteristics, including male sex, acquisition site, Pitt score, and Charlson Comorbidity Index, did not differ significantly between groups.

### Treatment options and outcomes

Patients in the comparator group received diverse combination regimens, most commonly amikacin (85.3%), tigecycline (66.0%), prolonged-infusion meropenem (52.6%), and, in nearly one-third of cases, colistin (Table 2). Detailed regimen combinations are provided in Table S2. The median time from index culture to initiation of active antimicrobial therapy was 2 days (IQR, 0–3) in the CZA group and 2 days (IQR, 1–4) in the comparator group (*P* < 0.001).

**TABLE 2:** Detailed composition of best available antimicrobial regimens in the comparator group (*n* = 156)^*a*^

Component of combination therapy	*n* (%)
Meropenem, prolonged infusion	82 (52.6)
Meropenem, standard infusion	38 (24.4)
Colistin	46 (29.5)
Tigecycline	103 (66.0)
Amikacin	133 (85.3)

^*a*^ Data are presented as *n* (%) unless otherwise indicated.

**Fig 2:**
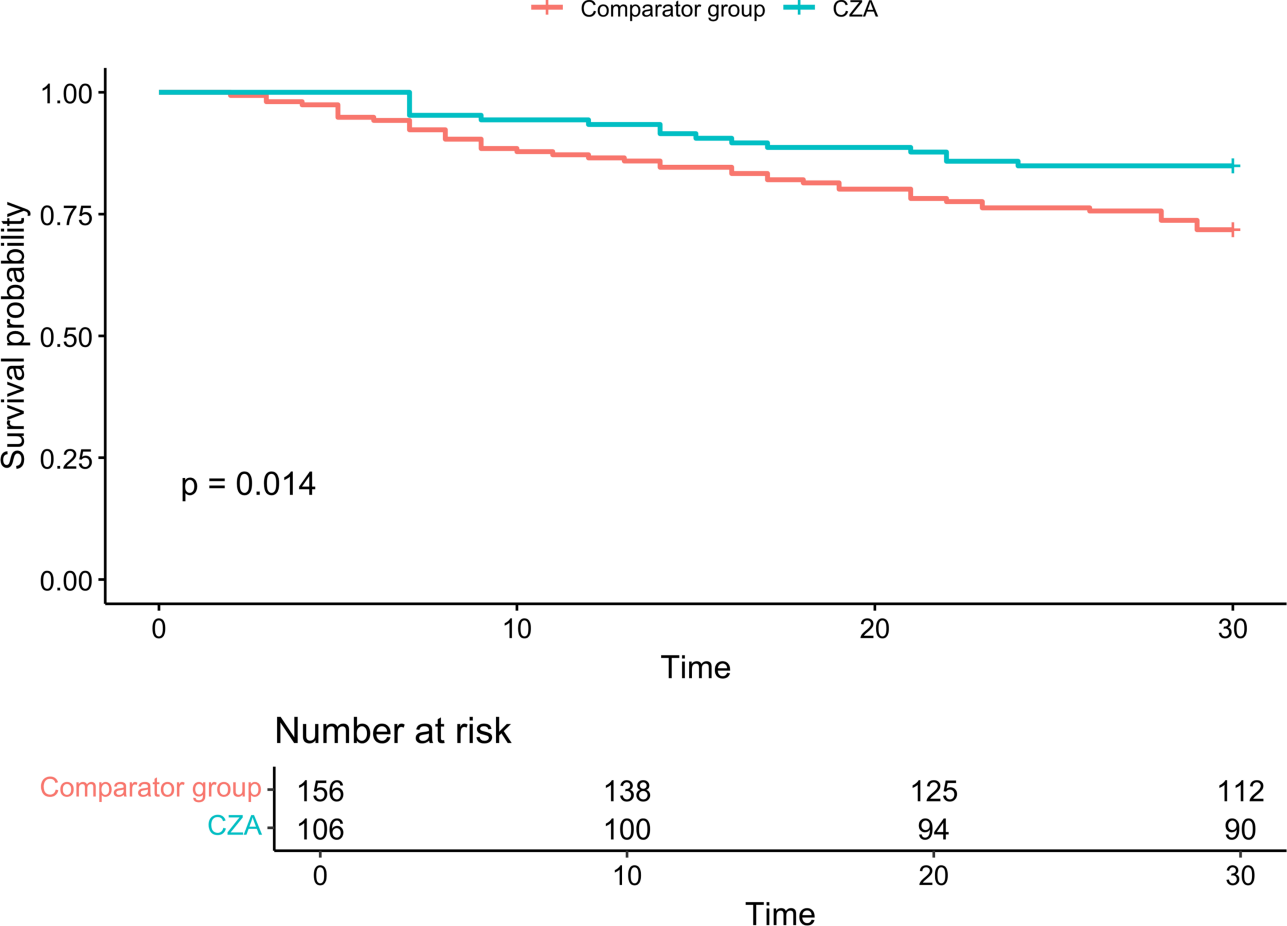
Comparison of 30-Day Kaplan–Meier survival curves between CZA and comparator groups. CZA, ceftazidime-avibactam.

Clinical outcomes are shown in Table 1. Thirty-day all-cause mortality was significantly lower in the CZA group (15.1%, 16/106) compared with the comparator group (28.2%, 44/156; *P* = 0.02). Similarly, 90-day mortality was reduced in the CZA group (26.4% vs 42.9%; *P* = 0.01). Persistent BSI occurred significantly less frequently in the CZA group (5.0% vs 34.2%; *P* < 0.001), whereas the incidence of recurrent BSI was similar between the groups (13.2% vs 12.2%; *P* = 0.96). Breakthrough infection or recurrence with acquired resistance occurred in three CZA recipients (2.8%). Kaplan–Meier analysis demonstrated significantly higher 30-day survival in the CZA group compared with the comparator group (*P* = 0.014; Fig. 2).

### Risk factors for 30-day mortality in KPC-producing Enterobacterales BSI

Univariate analysis identified variables with a *P*-value of less than 0.05, which were then considered for multivariate logistic regression (Table 3). Chronic liver disease and solid cancer were excluded from the model as they were already captured within the Charlson Comorbidity Index. Additionally, ICU admission at BSI onset was retained as an indicator of critical illness, as nearly all mechanically ventilated patients were admitted to the ICU.

**TABLE 3:** Risk factors associated with 30-day mortality in KPC-producing Enterobacterales bacteremia^*a*^^,^^*b*^

Variable	Univariate analysis			Multivariate analysis		
	Odds ratio	95% CI	*P*-value	Adjusted odds ratio	95% CI	*P*-value
Age	1.04	1.02–1.07	0.002			
CZA treatment	0.45	0.23–0.84	0.01	0.46	0.22–0.92	0.03
Initial culture						
Monomicrobial	1.00	–^*c*^	–			
Polymicrobial	2.49	1.03–5.84	0.04			
ICU care at index culture	2.76	1.42–5.32	0.003			
Pitt bacteremia score	1.51	1.32–1.75	<0.001	1.61	1.38–1.90	<0.001
Charlson comorbidity index	1.21	1.08–1.36	0.001	1.23	1.07–1.40	0.004
Solid organ transplant	0.45	0.21–0.92	0.04	0.22	0.08–0.54	0.002

^*a*^ CZA, ceftazidime–avibactam; CI, confidence interval.

^*b*^ Monomicrobial: initial culture showing only KPC-producing CPE; Polymicrobial: initial culture showing KPC-producing CPE along with one or more other organisms.

^*c*^ –, not applicable.

In multivariate analysis, higher Pitt bacteremia scores (adjusted odds ratio [aOR] 1.61, 95% CI, 1.38–1.90) and a higher CCI (aOR 1.23, 95% CI, 1.07–1.40) were independently associated with increased 30-day mortality. In contrast, receipt of CZA (aOR 0.46, 95% CI, 0.22–0.92) and SOT (aOR 0.22, 95% CI, 0.08–0.54) were independently associated with reduced mortality (Table 3).

In addition, propensity score matching was performed as a sensitivity analysis to adjust for baseline imbalances. The baseline characteristics of the propensity score-matched cohort are presented in Table S3. After matching, mortality occurred in 14 patients (19.4%) in the CZA group and in 22 patients (30.6%) in the comparator group. In the cohort, conditional logistic regression showed that CZA treatment tended to be associated with reduced odds of the outcome although the association was not statistically significant (OR, 0.56; 95% CI, 0.26–1.20; *P* = 0.14).

### Emergence of CZA resistance

Among the 106 patients treated with CZA, resistance emerged in three cases (2.8%) (Table 4). One patient with myelodysplastic syndrome developed a breakthrough infection during CZA treatment (at 19 days), while two others, both SOT recipients, experienced recurrent infections with acquired resistance. These patients had received CZA for 14 and 16 days, respectively, for their index BSI. Recurrent BSI with CZA-resistant *K. pneumoniae* occurred on days 27 and 97 following the completion of therapy.

**TABLE 4:** Clinical and microbiological characteristics of patients with emerging resistance to CZA^*a*^

Classification	No	Age/sex	Underlying diseases	Type of infection	30-day mortality	90-day mortality	Persistent BSI during initial BSI	Organism	Treatment after resistance	Time to acquire resistance from index BSI (day)	CZA MIC at index culture (mg/L)	CZA MIC at resistance (mg/L)
Breakthrough infection	1	31/M	Myelodysplastic syndrome	Intra-abdominal	Survived	Deceased	(+)	*K. pneumoniae*	Meropenem, amikacin, CZA	18	1	>8
Recurrent infection with acquired resistance	2	62/M	Liver transplant recipient	Biliary tract	Survived	Survived	(−)	*K. pneumoniae*	Meropenem, amikacin	27	0.25	>8
Recurrent infection with acquired resistance	3	49/M	Liver transplant recipient	Biliary tract	Survived	Survived	(−)	*K. pneumoniae*	Meropenem, amikacin, CZA	97	1	>8

^*a*^ CZA, ceftazidime–avibactam.

## DISCUSSION

This single-center retrospective cohort study conducted in South Korea, including 262 adults with KPC-producing Enterobacterales BSIs, found that CZA therapy was associated with significantly lower 30-day all-cause mortality and persistent BSI compared with best available agents. Multivariable analysis confirmed CZA as an independent predictor of survival. In bloodstream infections, resistance to CZA was infrequently observed, with only three patients (2.8%) developing resistant isolates, one of whom (0.9%) developed resistance during treatment.

Our findings align with a meta-analysis by Karampatakis et al., which reported significantly improved 30-day mortality with CZA compared with alternative treatments for carbapenem-resistant *K. pneumoniae* infections (23.2% vs 42.0%, odds ratio [OR] = 0.33, *P* < 0.001) (14). The lower overall mortality observed in our cohort compared with this meta-analysis may reflect several factors. First, ICU admission rates were lower in our cohort than in previous studies (21.7% vs 33%–53%) (15–17), and ICU admission is a well-established risk factor for worse outcomes (18). Second, the Pitt bacteremia score, a marker of acute illness severity, was lower in our study (median score 1 vs 4) (15, 16), with scores ≥4 associated with critical illness severity and increased mortality (19). Third, Falcone et al. reported that timely administration of effective antibiotics significantly improves clinical outcomes in patients with KPC-producing *K. pneumoniae* BSIs (20). In our study, the median time to CZA initiation was 2 days, whereas Tumbarello et al. documented a median of 7 days, potentially explaining the more favorable outcomes observed in our patients (21).

The rate of persistent BSI observed in our cohort is consistent with previous studies (15, 21). Tsolaki et al. reported microbiological eradication by day 10 in 94.3% (33/35) of patients receiving CZA, including 100% (18/18) of BSI cases (22). Similarly, Shields et al. found no persistent bacteremia among 13 patients treated with CZA, compared with 17.7% (17/19) in those receiving alternative treatment regimens (15). The low rate of persistent BSI observed in our study may reflect the high *in vitro* susceptibility of KPC-producing Enterobacterales to CZA in Korea; recent surveillance data reported 98.9% (184/186) of KPC-2-producing isolates were susceptible (9).

In our study, CZA use and SOT status were independent protective factors for 30-day mortality, whereas higher comorbidity burden and Pitt bacteremia scores were associated with increased risk. While the protective effect of CZA has been widely reported (16, 22, 23), the association with SOT status is less well described and is likely multifactorial. First, in a multicenter matched cohort of CRE cases, Boutzoukas et al. reported that SOT recipients had a lower 30-day mortality (14% vs 25% , *P* = 0.018) than non-SOT patients (24). In their cohort, infections included urinary tract (43%), respiratory (19%), bloodstream (17%), wound (12%), abdominal (6%), and other sources (3%), with 51% meeting infection criteria and groups matched on culture source. The authors suggested that younger age, lower acute illness severity, and earlier involvement of infectious diseases specialists, along with more frequent admission from home and a lower prevalence of malignancy, may have contributed to the improved outcomes. SOT patients in our cohort were significantly younger (Table S1), and at our center, SOT recipients are routinely co-managed by infectious diseases specialists, allowing timely consultation and optimization of antimicrobial therapy. Second, early initiation of appropriate treatment may have contributed to improved outcomes. In a study by Falcone et al., the administration of appropriate antibiotics within 24 h significantly reduced mortality in patients with KPC-producing *K. pneumoniae* BSI (20). Similarly, in our cohort, a higher proportion of SOT patients received appropriate antibiotic therapy within 24 h compared with non-SOT patients (Tables S1, 48.6% vs 35.8%, *P* = 0.058). Although this difference did not reach statistical significance, the trend suggests that timely intervention may have played a role in improving survival outcomes among SOT recipients.

In our cohort, CZA resistance was observed in 2.8% (3/106) of cases, with on-treatment resistance emerging in one patient (0.9%). Two additional patients (1.9%, 2/102) developed resistance upon recurrence. These findings are consistent with previous reports on CZA resistance in KPC-producing Enterobacterales BSI. Tumbarello et al. demonstrated *in vitro* CZA resistance rates of 1.9% (2/104) and 3.6% (14/391) in their respective cohorts, aligning with the results observed in our study (16, 23).

This study has several limitations. First, it was conducted at a single center employing a retrospective design, which may have introduced selection bias. Second, because the cohorts were not matched *a priori*, residual confounding cannot be fully excluded. In a propensity score-matched sensitivity analysis, the direction of effect remained consistent; however, limited common support and the small sample size resulted in imprecise estimates and loss of statistical significance. Third, the comparator cohort was historical; as such, temporal changes in infection-control practices, supportive care, and the indirect effects of the COVID-19 pandemic could have influenced outcomes. Fourth, the comparator group received heterogeneous combination regimens, preventing disentanglement of the independent effects of individual agents and introducing confounding by indication. Fifth, uncertainty regarding the true *in vitro* activity of colistin (in the absence of reference broth microdilution) and the lack of tigecycline susceptibility data limit the interpretation of outcomes associated with regimens containing these agents. Finally, because we were unable to perform genomic characterization of the three CZA-resistant cases, we could not determine whether these episodes represented acquired resistance or recurrence, nor could we define the sequence types or specific resistance mechanisms of the isolates.

In conclusion, CZA therapy was associated with significantly lower 30-day mortality and persistent bacteremia compared with best available agents in patients with BSI caused by KPC-producing Enterobacterales. Given the previously limited therapeutic options available in South Korea, these findings support CZA as a highly effective first-line treatment in this setting. Further prospective, multicenter studies are warranted to validate our findings and to monitor the emergence of resistance with broader clinical use.

## SUPPLEMENTAL MATERIAL

Supplemental tables spectrum.02819-25-s0001.docx Tables S1 to S3.

ASM does not own the copyrights to Supplemental Material that may be linked to, or accessed through, an article. The authors have granted ASM a non-exclusive, world-wide license to publish the Supplemental Material files. Please contact the corresponding author directly for reuse.
