# Supplementary material for: Comparison of outcomes of KPC-producing Enterobacterales bloodstream infections treated with ceftazidime-avibactam and other conventional antibiotics: retrospective single-center study
Source: Microbiol Spectr. 2026 Mar 12;14(4):e02819-25. doi: 10.1128/spectrum.02819-25 (PMC13055243; doi:10.1128/spectrum.02819-25)
Supplement: Supplemental tables — Tables S1 to S3. [file spectrum.02819-25-s0001.docx]

**Supplementary Material**

**Table S1.** Comparison of clinical data between solid organ transplant (SOT) and non-SOT patients

|  | **SOT (n=72)** | **Non-SOT (n=190)** | **p-value** |
| --- | --- | --- | --- |
| **Age (years), median (IQR)** | 61.0 (55.0–64.5) | 66.0 (57.0–71.0) | < 0.001 |
| **Male sex** | 43 (59.7) | 108 (56.8) | 0.78 |
| **Acquisition site** |  |  | 0.92 |
| Community | 0 (0) | 2 (1.1) |  |
| Healthcare-associated community | 11 (15.3) | 27 (14.2) |  |
| Healthcare | 61 (84.7) | 161 (84.7) |  |
| **ICU at index culture** | 23 (31.9) | 28 (14.7) | 0.003 |
| **Hemodialysis at index culture** | 16 (22.2) | 16 (8.4) | 0.005 |
| **CRRT at index culture** | 11 (15.3) | 12 (6.3) | 0.04 |
| **ECMO at index culture** | 3 (4.2) | 1 (0.5) | 0.06 |
| **Pitt bacteremia score, median (IQR)** | 1.0 (0.0–3.0) | 1.0 (0.0–2.0) | 0.34 |
| **Charlson comorbidity index, median (IQR)** | 5.5 (5.0–7.0) | 6.0 (4.0–8.0) | 0.60 |
| **CZA use** | 32 (44.4) | 74 (38.9) | 0.50 |
| **Time to appropriate treatment, median (IQR)** | 2.0 (1.0–3.0) | 2.0 (1.0–4.0) | 0.08 |
| **Appropriate antibiotic use within 24 hours** | 35 (48.6) | 68 (35.8) | 0.058 |
| **30-day mortality** | 10 (13.9) | 50 (26.3) | 0.049 |

Data are presented as *n* (%) unless otherwise indicated.
Abbreviation: CZA ceftazidime–avibactam; IQR, interquartile range; SOT, solid organ transplant.

**Table S2.** Characteristics of antibiotic treatment regimens

| **Component therapy** | **n (%)** |
| --- | --- |
| Monotherapy | 19 (12.2) |
| Combination therapy | 137 (87.8) |
| Specific regimen |  |
| Colistin-based | 7 (4.9) |
| Colistin with aminoglycoside | 96 (61.5) |
| Aminoglycoside-based | 39 (25.0) |
| Tigecycline-based | 9 (5.8) |
| Others | 5 (3.2) |

Colistin-based: Defined as colistin monotherapy or colistin in combination with antibiotics other than amikacin.

Aminoglycoside-based: Defined as amikacin monotherapy or amikacin in combination with antibiotics other than colistin.

Colistin with aminoglycoside: Defined as antibiotic combinations containing both colistin and aminoglycoside.

Tigecycline-based: Defined as tigecycline monotherapy or tigecycline in combination with antibiotics other than colistin and aminoglycosides.

Others: Defined as meropenem monotherapy or other regimens not included in the above categories.

**Table S3.** Baseline characteristics after propensity score matching

|  | **CZA group(n=72)** | **CPE-specific agent group (n=72)** | **SMD** |
| --- | --- | --- | --- |
| **Age (years), mean (SD)** | 62.0 (12.3) | 62.7 (13.6) | 0.06 |
| **Male sex** | 40 (55.6) | 39 (54.2) | 0.01 |
| **Ward** |  |  |  |
| Medical | 39 (54.2) | 35 (48.6) | 0.05 |
| Surgical | 18 (25.0) | 22 (30.6) | 0.05 |
| ICU | 15 (20.8) | 15 (20.8) | 0.00 |
| **Acquisition site** |  |  |  |
| Community | 1 (1.4) | 1 (1.4) | 0.00 |
| Healthcare-associated community | 11 (15.3) | 10 (13.9) | 0.01 |
| Healthcare | 60 (85.3) | 61 (84.7) | 0.01 |
| **Previous CPE bacteremia** | 5 (6.9) | 4 (5.6) | 0.01 |
| **CPE colonization at index culture** | 57 (79.2) | 57 (79.2) | 0.00 |
| **Site of infection** |  |  |  |
| Intra-abdominal | 27 (37.5) | 29 (40.3) | 0.02 |
| Biliary tract | 29 (40.3) | 26 (36.1) | 0.04 |
| Catheter-related | 7 (9.7) | 3 (4.2) | 0.05 |
| Pneumonia | 5 (6.9) | 8 (11.1) | 0.04 |
| Skin and soft tissue | 1 (1.4) | 1 (1.4) | 0.00 |
| Urinary tract | 3 (4.2) | 4 (5.6) | 0.01 |
| Primary bacteremia | 0 (0) | 1 (1.4) | 0.01 |
| **Pitt bacteremia score, mean (SD)** | 1.6 (1.9) | 1.6 (2.4) | 0.02 |
| **Charlson comorbidity index, mean (SD)** | 5.9 (2.4) | 6.1 (2.7) | 0.08 |
| **Comorbidities** |  |  |  |
| Chronic liver disease | 29 (40.3) | 28 (38.9) | 0.01 |
| Hematological malignancy | 24 (33.3) | 23 (31.9) | 0.01 |
| Neutropenia | 21 (29.2) | 21 (29.2) | 0.00 |
| Diabetes mellitus | 22 (30.6) | 25 (34.7) | 0.04 |
| Solid organ transplant | 20 (27.8) | 23 (31.9) | 0.04 |
| Solid cancer | 27 (37.5) | 27 (37.5) | 0.00 |
| Chronic kidney disease | 11 (15.3) | 12 (16.7) | 0.01 |
| Cerebrovascular accident | 3 (4.2) | 3 (4.2) | 0.00 |
| Hematologic stem cell transplant | 24 (33.3) | 23 (31.9) | 0.01 |
| Chronic pulmonary disease | 2 (2.8) | 5 (6.9) | 0.04 |
| Chronic heart failure | 2 (2.8) | 1 (1.4) | 0.01 |
| **Mechanical ventilation at index culture** | 15 (20.8) | 15 (20.8) | 0.00 |
| **CRRT at index culture** | 7 (9.7) | 5 (6.9) | 0.03 |
| **ECMO at index culture** | 1 (1.4) | 1 (1.4) | 0.00 |
| **Outcome** |  |  |  |
| 30-day mortality | 14 (19.4) | 22 (30.6) |  |

Data are presented as *n* (%) unless otherwise indicated.

Abbreviations: CPE, carbapenemase-producing Enterobacterales; CRRT, continuous renal replacement therapy; CZA, ceftazidime–avibactam; ECMO, extracorporeal membrane oxygenation; ICU, intensive care unit; SD, standard deviation; SMD, Standardized mean difference.
